# Evaluation of Sterility of Saline Formulations Manufactured for Wound Care in Veterinary Practice

**DOI:** 10.3390/vetsci12050431

**Published:** 2025-04-30

**Authors:** Madyson Marcolina, Zoë J. Williams, Dean Hendrickson, Lynn M. Pezzanite

**Affiliations:** Department of Clinical Sciences, Colorado State University, Fort Collins, CO 80523, USA

**Keywords:** wound, dressings, hypertonic, saline

## Abstract

The discontinuation of commercially available saline and hypertonic saline wound dressings for the veterinary market has restricted options available to veterinary practitioners treating contaminated and infected wounds. Clinicians may manufacture their own homemade solutions in clinics or field settings to treat equine or livestock species; however, information is limited on whether autoclave sterilization is necessary or sufficient to eliminate bacterial growth in isotonic and concentrated salt solutions and how long they may subsequently be stored prior to use. The purpose of this study was to assess sterility of saline (0.9%) and hypertonic saline (20%) solutions manufactured three ways (1—autoclaved glass bottle that was autoclaved again following solution preparation; 2—autoclaved glass bottle, not autoclaved again following preparation; 3—non-autoclaved plastic bottle, not autoclaved following preparation). Solutions were stored two different ways (1—solution in sealed bottle or 2—soaked gauze in vacuum-sealed plastic packets). Products were assessed for bacterial growth at four time points (baseline, one week, one month, six months). At each time point, samples of each solution were plated on Luria–Bertani (LB) agar plates and assessed for bacterial growth at 24 h. Vacuum-sealed soaked gauze was placed in antibiotic-free growth media for 24 h, and then media were plated on LB agar plates and assessed for bacterial growth at 24 h. If bacterial growth was detected, qualitative culture with sensitivity was performed to identify bacterial isolates. No bacterial growth was detected in stored solutions for any preparation method, concentration or time point assessed. Bacterial growth was detected from 0.9% saline-soaked gauze at 1 week, 1 month and 6 months in all container types for at least one time point. Bacterial culture revealed *Ralstonia*, *Bacillus*, *Sphingomonas* and *Staphylococcus* species. Environmental controls (water, containers, salt, biosafety cabinet and benchtop) were submitted for culture to identify the source of contamination, yielding light mixed growth from tap water and no growth from any other locations. These findings provide clinicians with practical information to guide preparation and storage of homemade saline-based products for wound care.

## Simple Summary

Hypertonic saline products have been effectively applied in early stages of wound healing to reduce bacterial load in contaminated and infected wounds. Commercially available saline and hypertonic saline products (solutions, treated gauze) have been recently discontinued in the veterinary market, limiting options available to practitioners for wound care. Therefore, the goal of this work was to assess the sterility of homemade saline and hypertonic saline solutions and treated gauze produced under different conditions and stored for up to six months. These findings are intended to provide clinicians with actionable information on options for wound care in veterinary species.

## 1. Introduction

Increased recognition of the role that biofilms play in the persistence of chronically infected wounds in equine practice highlights the need for further development of local wound care strategies [1,2,3]. A chronic wound has been described as one that has delayed, interrupted or stalled healing with slow progression through healing phases [4]. Although traditional bacterial culturing techniques are considered inadequate to comprehensively identify bacterial species associated with biofilms [5], a chronic non-healing wound has been considered suggestive of biofilm infection, provided that other underlying pathologies such as ischemia have been excluded [4]. While multiple definitions of biofilms have been posed [6,7,8], recent articles have defined them as structured communities of microbes with genetic diversity and variable gene expression that create behaviors and defenses used to produce unique (chronic) infections [4]. This is compounded by mounting evidence for bacterial resistance in veterinary practice [9,10,11,12,13,14]. Lavage, debridement and topical wound care strategies are effective ways to reduce bacterial load within wounds and minimize necrotic tissue towards rapid and cosmetic wound healing as systemic antibiotics may not penetrate infected tissues locally [15].

Multiple debridement methods to selectively kill bacteria while minimizing local cytotoxicity are available, including autolytic, biological, enzymatic/chemical, mechanical or sharp techniques. These methods may be applied alone or in conjunction to alter wound classification from infected or contaminated to clean-contaminated or clean. Recent consensus documents in human wound care have emphasized the value of local debridement with biofilm-based wound care (BBWC) strategies to manage infected and chronically non-healing tissues [8,16,17,18]. Briefly, BBWC treatment recommends management of biofilms in three stages: (1) physical disruption/debridement of the biofilm, (2) topical treatments to prevent or delay reformation and (3) repeated therapy of steps 1 and 2 until full resolution is achieved [19]. These strategies emphasize the necessity for repeated physical disruption of the biofilm structure to disrupt the matrix and remove devitalized tissue that serves as a source of nutrients to the microbes. These techniques both physically remove bacteria to reduce overall bioburden and further enhance the susceptibility of bacteria within biofilm communities to antimicrobial therapies [20].

Topical application of hypertonic (20%) saline dressings is considered an inexpensive, non-invasive and practical option to achieve local chemical debridement, with advantages over isotonic (0.9%) saline solutions during initial stages of wound dressing [21,22,23]. The primary mechanism of action behind hypertonic dressings is performed by achieving an osmotic gradient, drawing fluid from tissues and removing exudate and debris, effectively reducing contamination in wounds with minimal damage to local tissues [24,25,26]. Hypertonic saline has also been shown to debride heavily exudative wounds in combination with negative pressure wound therapy in humans, reducing infection rates and the need for surgical debridement [26]. However, while hypertonic saline has been demonstrated to be superior in the initial stages of healing, continued hypertonic dressings contribute to negative outcomes once granulation tissue forms, contributing to the desiccation of healing granulation tissue when compared to normal saline [21]. Mechanical debridement through saline lavage, woven gauze or wet-to-wet or wet-to-dry dressings may be integrated in conjunction with chemical debridement based on the stage of healing [25]. Saline lavage alone has been shown to be effective in reducing bacterial counts in an infected wound, with no detrimental effect on wound healing such as that induced by antiseptics, which are more appropriately applied around but not in wounds [27,28,29,30].

However, recent world-wide discontinuation of commercially available saline-based wound dressings for the veterinary market has restricted options accessible to practitioners to purchase and driven demand for alternative economical medical solutions. Current shortages in saline-based supplies for lavage and debridement and economic limitations pose challenges to veterinary practitioners and animal caretakers, particularly in resource-limited and rural settings, where access to medical supplies is constrained. The evaluation of substitute products that may be consistently manufactured could provide alternatives in these environments and potentially have broader applications to inform potential use in human medicine in austere environments.

The aim of this study was to assess the sterility of homemade saline solutions manufactured under different conditions and packaging techniques and stored for time periods up to six months. The identification of practices (e.g., autoclave sanitization) necessary to minimize additional wound contamination with the introduction of stored saline-based wound care products will provide practitioners with practical recommendations to enhance the accessibility and safety of saline treatments in contexts where commercial products are unavailable or unaffordable.

## 2. Materials and Methods

**Figure 1:**
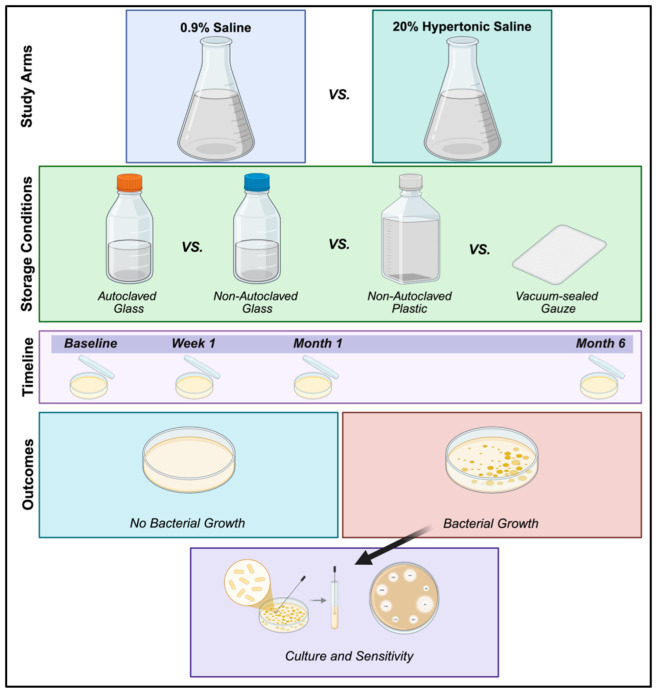
Overview of study design. Williams, Z. (2025). Created in BioRender. https://BioRender.com/s34o929.

***Study overview***—saline (0.9%) and hypertonic saline (20%) solutions were manufactured on the benchtop in the laboratory using three different containers and methods of sterilization: Group 1—autoclaved glass bottles that were autoclaved again following solution preparation; Group 2—autoclaved glass bottles that were not autoclaved again following preparation and addition of saline; and Group 3—non-autoclaved plastic bottles that were not autoclaved following preparation and addition of saline. Solutions were then stored two different ways: (1) solution in sealed bottle or (2) soaked gauze placed immediately into vacuum-sealed plastic packets in the laminar flow biosafety cabinet hood. Solutions and plastic packets were then stored on the laboratory benchtop at room temperature and subjectively assessed for bacterial growth at four different time points (baseline, one week, one month, six months). A study overview is summarized in Figure 1.

***Solution and packet preparation***—saline and hypertonic saline solutions were prepared on the benchtop using iodized table salt (Morton Salt Food Company, Chicago, IL, USA). Saline (0.9%) solutions were prepared by a combination of 9 g salt in 1000 mL tap water. Hypertonic saline (20%) solutions were prepared by a combination of 200 g salt in 1000 mL tap water. Tap water was used to replicate the scenario commonly encountered by practitioners or livestock owners in preparing wound dressing solutions for use in the field. To prepare the Groups 1 and 2 solutions, freshly autoclaved glass bottles were used (autoclave settings for gravity, 15 min, at 121 °C). To prepare the Group 1 solution, the glass bottles were then re-autoclaved again following the addition of salt to the solution in the bottle, using the same autoclave settings).

**Figure 2:**
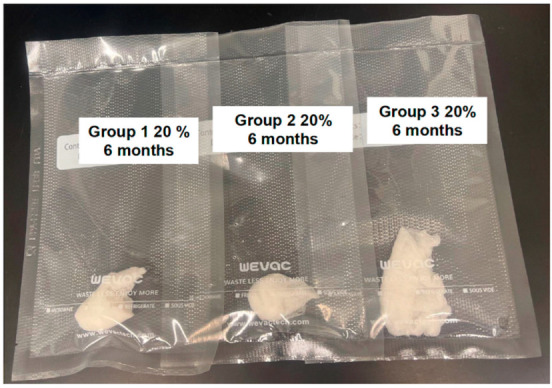
Soaked gauze in vacuum-sealed commercially available plastic packets packaged using a food vacuum sealer for storage. Treatment groups from left to right: Group 1—autoclaved glass bottles that were autoclaved again following solution preparation; Group 2—autoclaved glass bottles that were not autoclaved again following preparation and addition of saline; and Group 3—non-autoclaved plastic bottles that were not autoclaved following preparation and addition of saline.

Following preparation of solutions, samples of each solution were taken from bottles within the laminar flow biosafety cabinet hood. Sterile 4 × 4 inch gauze pads (Covidien LLC, Mansfield, MA, USA) were soaked in each solution and vacuum-sealed into commercially available plastic packets (Wevac vacuum sealer bags, Weston commercial grade, BPA free, heavy duty kitchen bags, Wevac Technology Co., Hong Kong, China) using a commercially available food vacuum sealer (Food Vacuum Sealer Machine, 65 Kpa Compact Vacuum Food Preservation System LED Indicator, Mesliese, Bellevue, WA, USA) within the biosafety cabinet (Figure 2). Solutions and packets were then maintained at room temperature on the laboratory bench until bacterial culture at the designated time point.

***Bacterial culture***—at each time point (baseline immediately following preparation of solutions, one week, one month, six months), samples of each solution were plated in quadruplet on Luria–Bertani (LB) agar plates and assessed for bacterial growth at 24 h. Vacuum-sealed packets were opened in the biosafety cabinet, and gauze was placed in antibiotic-free growth media (Dulbecco’s Modified Eagle’s Medium (DMEM) (Corning Life Sciences, Durham, NC, USA) with 1000 mg/L glucose and 10% fetal bovine serum (FBS) (PEAK Serum, Wellington, CO, USA), 1M HEPES) for 24 h, and then media were plated on LB agar plates in quadruplet and assessed subjectively for bacterial growth at 24 h. Packets were opened individually at each time point (i.e., swabs were not returned to the vacuum-sealed packet for evaluation at later time points). Negative controls (sterile saline) and positive controls (methicillin-resistant *Staphylococcus aureus* strain ATCC 25923) were plated additionally at each time point. If bacterial growth was detected following 24 h in culture, samples were submitted to the Colorado State University Diagnostic Laboratory for qualitative culture with sensitivities to identify bacterial isolates. Environmental control samples (tap water, containers, salt, hood and benchtop) were also submitted for bacterial culture and sensitivity to determine the source of contamination in instances where bacterial growth occurred. Environmental controls were obtained via sterile culture swabs (BD BBL CultureSwab Plus Specimen Collection and Transport System) and then swabbed onto plates to assess for bacterial growth. Positive control samples of *Staphylococcus aureus* with known bacterial sensitivity were submitted at each time point and with environment control samples.

***Data analysis***—bacterial culture plates were visually assessed for bacterial colony growth at each time point (baseline, one week, one month, six months), which was recorded descriptively (yes or no if bacteria growth was present). In instances where bacterial growth was noted, qualitative bacterial culture and sensitivities were performed and reported descriptively. Figure generation was created in Biorender. Williams, Z. (2025) https://BioRender.com/s34o929.

## 3. Results

**Figure 3:**
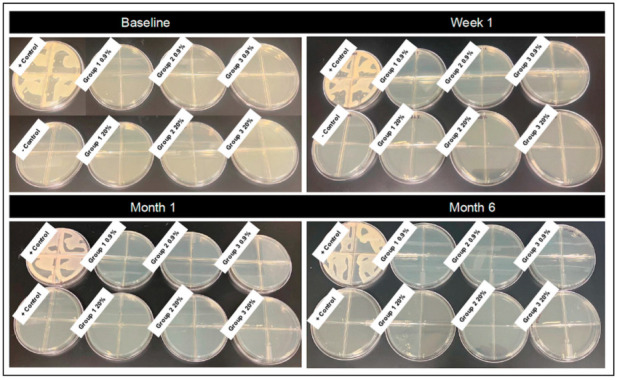
**Representative images of bacterial plates following culture of saline solutions**. Isotonic (0.9%) and hypertonic saline (20%) solutions were plated at four time points (baseline, one week, one month, six months). Treatment groups: Group 1—autoclaved glass bottles that were autoclaved again following solution preparation; Group 2—autoclaved glass bottles that were not autoclaved again following preparation and addition of saline; and Group 3—non-autoclaved plastic bottles that were not autoclaved following preparation and addition of saline.

**Figure 4:**
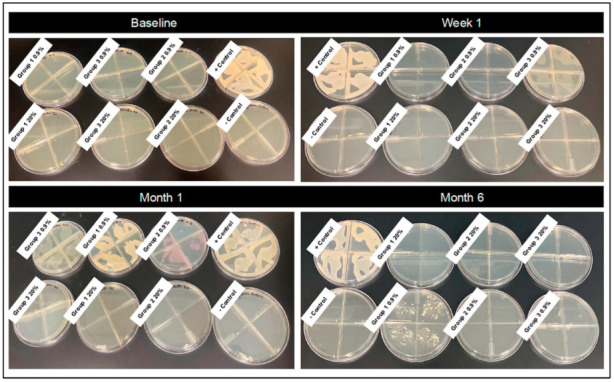
**Representative images of bacterial plates following culture of soaked gauze.** Isotonic saline (0.9%) and hypertonic saline (20%) saline-soaked gauze was plated at four time points (baseline, one week, one month, six months). Treatment groups: Group 1—autoclaved glass bottles that were autoclaved again following solution preparation; Group 2—autoclaved glass bottles that were not autoclaved again following preparation and addition of saline; and Group 3—non-autoclaved plastic bottles that were not autoclaved following preparation and addition of saline.

Bacterial growth was not detected in stored solutions for any preparation method (Groups 1, 2 or 3), saline concentration (0.9%, 20%) or time point (baseline, one week, one month, six months) assessed (Table 1). Bacterial growth was detected from gauze sealed in vacuum-sealed plastic packets at the 1-week time point for Group 3 0.9%, at the 1-month time point for all 0.9% treatments (Groups 1, 2 and 3) and at the 6-month time point for Group 1 0.9% treatment. Gauze in vacuum-sealed packets at the 6-month time point had dried out for all three treatments assessed, making assessment of growth not possible. No bacterial growth was detected from gauze soaked in 20% hypertonic saline for any time point assessed (Figure 3 and Figure 4).

The results of qualitative bacterial cultures to identify bacterial species cultured from gauze are summarized in Table 2. Culture revealed *Ralstonia*, *Bacillus*, *Sphingomonas and Staphylococcal* species. Environmental controls (tap water, containers, salt, biosafety cabinet and benchtop) were submitted for culture to identify potential sources of contamination, yielding light mixed growth in tap water of at least four species, consistent with what was identified at the one-week and one-month time points. Resistance patterns for each species are indicated in Table 3. No growth was identified from cultures submitted from any of the other environmental controls.

## 4. Discussion

This study indicated that normal (0.9%) and hypertonic (20%) saline solutions can be stored for up to six months at room temperature in sealed bottles without evidence of bacterial growth based on routine culture methods. However, saline (0.9%)-soaked gauze stored in commercially available vacuum-sealed packets did yield bacterial growth as early as one week when made in non-autoclaved plastic containers. Hypertonic saline (20%)-soaked gauze stored in vacuum-sealed containers did not yield growth at any time point. At the six-month time point, all soaked gauze treatments were desiccated upon opening, indicating clinical utility may be diminished if stored for long periods in this manner.

The implementation of normal and hypertonic saline lavage and dressings represents practical and inexpensive wound care options that can be applied in rural or austere settings to improve wound management [21,22,23]. Both tonicities of saline were evaluated here as they have been shown to be most appropriately applied at different stages of wound healing. Normal and hypertonic saline may be used to improve outcomes and speed healing in contaminated wounds, with saline lavage as a sole therapy demonstrated to reduce bacterial counts without risk of local cytotoxicity described with topical antiseptics [27,28,29,30]. Hypertonic saline applied temporarily has further been shown to be efficacious as a debridement dressing during initial stages of management, exerting an enhanced effect to remove exudate and debris through osmotic action [24,25,26]. However, prolonged exposure to hypertonic saline contributes to negative outcomes past the debridement stage, inhibiting normal granulation tissue formation during contraction and epithelialization phases [21]. The findings of this study are intended to inform methods of production for both saline concentrations to facilitate the implementation of the appropriate solution based on the stage of wound healing. Outcomes indicate that while both solutions in sealed bottles may be usable for up to six months, normal saline-soaked gauze stored in sealed packets has a limited shelf life relative to hypertonic saline, and all gauze prepared in this manner was dried at six months, limiting use to that time frame.

Bacterial species identified included *Ralstonia*, *Bacillus*, *Sphingomonas and Staphylococcal* species in saline-soaked gauze packets as early as one week for normal saline treatments. Swabs of environmental controls (tap water, containers, salt, biosafety cabinet and benchtop from the bacterial laboratory) were submitted to identify potential sources of contamination, revealing low-level mixed microbiota growth from tap water, indicating the initial water used to make solutions was potentially the source of contamination and that bacterial growth was not inhibited sufficiently to prevent subsequent growth on agar plates in normal saline solutions. However, growth was not detected within the isotonic solutions in sealed containers. Gauze within the vacuum-sealed packets was desiccated by six months, suggesting the packet and sealing device employed here did not achieve an entirely airtight seal, which could conceivably allow for additional environmental contamination of the gauze inside. Therefore, whether the source of contamination was in fact the tap water initially used or through additional environmental contamination within the laboratory itself that was not picked up by routine screening cannot be discerned from the methods as described. However, the conclusion that the potential source of contamination could be from tap water does align with the previous literature, particularly regarding *Ralstonia* and *Sphingomonas* species, which have been implicated in contamination and infections associated with water and saline sources in medical settings worldwide [31,32,33,34,35,36,37,38]. Further evaluation of the importance of the initial water source (e.g., comparison to well or rainwater) in the shelf life of saline-soaked gauze relative to other parameters assessed (e.g., autoclaving, packaging method) are warranted.

Limitations to study design and caveats to conclusions drawn are worthy of mention. Although this study evaluated manufacturing of solutions in three storage containers that would be commonly available to practitioners, it is acknowledged that in field settings other types of containers may be used. Only one water source (tap water) was used in solution preparation, meaning that microbial loads from different sources, such as well or rainwater, were not accounted for here. Furthermore, solutions were made for this study in a relatively aseptic benchtop laboratory environment with limited potential for the introduction of contamination, which may not represent the environment necessitated for production by ambulatory clinicians or producers. Soaked gauze in packets was dehydrated at six months for all treatments assessed, limiting the ability to perform and assess viable bacterial cultures at the latest time point assessed. These findings suggest that while the vacuum sealing of gauze may be sufficient to maintain hydration for limited time points, the techniques employed here did allow for some air to enter the packets over time, leading to desiccation by six months. Further investigation of additional time points or alternate vacuum-sealing devices between months one and six to determine how long soaked gauze in packets remains moist and therefore clinically applicable represents a future study direction. The limitations of routine bacterial culture and sensitivity as the primary outcome parameter here are acknowledged, and additional assessments for anaerobic bacterial cultures or fungi were not performed. Furthermore, the functional antimicrobial activity of solutions developed here to effectively reduce bacterial colony counts in bacterial killing assays was outside the scope of this study.

Future studies would address these limitations by expanding the scope of bacterial testing performed to include a wider range of environmental and clinically relevant pathogens. Additional studies to evaluate homemade solutions prepared under non-sterile conditions to assess potential contamination risk would inform preparation in field settings. Further assessment of different water sources on sterility may impact outcomes, as variations in local microbial compositions may influence the efficacy of hypertonic saline solutions. Finally, an extended study assessing time points between one and six months for soaked gauze and beyond six months would be valuable to determine the true maximum shelf life of homemade normal and hypertonic saline solutions, providing further guidance on safe storage and use in veterinary and human medicine.

## 5. Conclusions

This study assessed the sterility of homemade saline and hypertonic saline solutions and treated gauze produced under different conditions and stored for up to six months. Outcomes support that solutions of both normal and hypertonic salt concentrations may be stored in closed containers up to six months without bacterial growth. However, normal saline-soaked gauze stored in vacuum-sealed packets demonstrated bacterial growth as early as one week following packaging, while hypertonic saline-soaked gauze did not have detectable growth at up to one month in any container assessed. Further investigation of methods to store soaked gauze to inhibit bacterial growth and reduce dehydration at later time points (i.e., up to six months) is warranted. These findings are intended to provide clinicians with actionable information on alternatives for topical wound care dressings in veterinary medicine as well as remote healthcare settings, including disaster response or military field medicine scenarios, where cost-effective, easily prepared antiseptic solutions are critically needed.

## Figures and Tables

**Table 1:** **Summary of bacterial culture results for saline and hypertonic saline solutions.** No growth was noted from either 0.9% or 20% solutions in any container at any time point. Treatment groups: Group 1—autoclaved glass bottles that were autoclaved again following solution preparation; Group 2—autoclaved glass bottles that were not autoclaved again following preparation and addition of saline; and Group 3—non-autoclaved plastic bottles that were not autoclaved following preparation and addition of saline. NG = no growth; N/A = not applicable.

	Baseline	1 week	1 month	6 months	Qualitative Culture Result
**Group 1**	NG	NG	NG	NG	N/A
**Group 2**	NG	NG	NG	NG	N/A
**Group 3**	NG	NG	NG	NG	N/A

**Table 2:** **A summary of bacterial culture results for 0.9% saline-soaked gauze.** The findings indicate that growth occurred at every time point following baseline in at least one container but was seen at the earliest time point (one week) in the non-autoclaved plastic container. No growth was seen in the 20% hypertonic saline solution-soaked gauze at any time point. Treatment groups: Group 1—autoclaved glass bottles that were autoclaved again following solution preparation; Group 2—autoclaved glass bottles that were not autoclaved again following preparation and addition of saline; and Group 3—non-autoclaved plastic bottles that were not autoclaved following preparation and addition of saline. NG = no growth.

	Baseline	1 week	1 month	6 months	Qualitative Culture Result
**Group 1**	NG	NG	growth	growth	1 month *Bacillus species*
					6 months *Staphylococcus aureus*
**Group 2**	NG	NG	growth	NG	1 month *Spingomonas, Bacillus species*
**Group 3**	NG	growth	growth	NG	1 week *Ralstonia species*
					1 month *Ralstonia species*

**Table 3:** **A summary of bacterial culture sensitivities results for 0.9% saline-soaked gauze.** The findings indicate that growth occurred at every time point following baseline in at least one container but was seen at the earliest time point (one-week) in the non-autoclaved plastic container.

Bacteria	Culture Sensitivities
***Ralstonia* spp.**	amikacin (MIC ≥ 64 week 1; MIC ≥ 32 month 1); amoxicillin/clavulanic acid (MIC ≥ 32 week 1); ampicillin (MIC ≥ 32 week 1);
	chloramphenicol (MIC ≥ 64 week 1; MIC ≥ 32 month 1); cefazolin (MIC ≥ 16 month 1); ceftazidime (MIC ≥ 32 month 1);
	ceftiofur (MIC ≥ 4 month 1); gentamicin (MIC ≥ 8 month 1); penicillin (MIC ≥ 8 month 1); ticarcillin/clavulanic acid (MIC ≥ 64 month 1)
***Bacillus* spp.**	no resistances noted (month 1)
***Sphingomonas* spp.**	cefazolin (MIC ≥ 16 month 1) and penicillin (MIC ≥ 2 month 1)
***Staphylococcus aureus***	penicillin (MIC 0.06 month 6)

## Data Availability

Data contained within the article.

## Abbreviations

The following abbreviations are used in this manuscript:

LB	Luria–Bertani
AUTO	Autoclaved glass bottle treatment group
NON-AUTO	Non-autoclaved glass bottle treatment group
PLASTIC	Non-autoclaved plastic bottle treatment group
NG	No growth
